# Injectable Thermoresponsive Hydrogels for Antibacterial Applications: Design Strategies, Antibacterial Mechanisms, and Biomedical Applications

**DOI:** 10.3390/ma19194093

**Published:** 2026-09-24

**Authors:** Jun-Ya Wang, Juan Sun, Yang Zhou

**Affiliations:** 1 School of Biological & Chemical Engineering, Zhejiang University of Science & Technology, Hangzhou 310023, China; 2 Laboratory of Advanced Theranostic Materials and Technology, Ningbo Institute of Materials Technology and Engineering, Chinese Academy of Sciences, Ningbo 315300, China; 3 Ningbo Cixi Institute of Biomedical Engineering, Cixi 315300, China

**Keywords:** thermoresponsive hydrogel, injectable, antibacterial, drug delivery, biofilm, infected wound

## Abstract

Injectable thermoresponsive hydrogels provide flowable precursors that form local depots on warming, offering a route to sustained antibacterial delivery in irregular tissue defects. This narrative review examines how thermal gelation, carrier architecture, and anatomical conditions jointly determine antibacterial exposure. It distinguishes polymer phase separation from macroscopic gelation, injectable precursors from topical and instilled formulations, and bacterial killing from biofilm and host-repair outcomes. Representative studies are compared by composition, delivery, thermal behavior, release conditions, microbiological endpoints, and animal model. Three design considerations emerge: gelation must occur within a practical administration window; drug availability depends on both the carrier and its local residence; and the required balance of retention, clearance, and tissue compatibility differs across wounds, infected bone defects, periodontal pockets, and ocular surfaces. Direct loading, particle-in-gel depots, contact-active components, and light-mediated treatments address different parts of this problem and require distinct efficacy and safety measurements. Current evidence supports preclinical antibacterial and regenerative applications, while heterogeneity in exposure and outcome measurements limits comparison across platforms. Progress toward clinical use depends on connecting formulation manufacture and material fate with local antibacterial exposure and indication-specific outcomes.

## 1. Introduction

Bacterial infections remain a major threat to public health worldwide. Although widespread antibiotic use has substantially controlled infectious diseases, it has also accelerated the dissemination of antimicrobial resistance. A global analysis estimated 1.27 million deaths attributable to bacterial antimicrobial resistance (95% uncertainty interval [UI], 0.911–1.71 million) and 4.95 million associated deaths (95% UI, 3.62–6.57 million) in 2019 [1]. Clinically prevalent pathogens, including methicillin-resistant *Staphylococcus aureus* (MRSA), *Pseudomonas aeruginosa*, and *Acinetobacter baumannii*, can resist antibiotics through reduced permeability, active efflux, target modification, and drug inactivation [2]. Biofilm-associated tolerance additionally involves matrix–drug interactions and physiological heterogeneity, contributing to persistent infection [3,4]. Against this background, the development of new antibiotics alone is unlikely to address complex infections adequately; optimizing local delivery and lesion retention has therefore become an important complementary strategy [5,6].

Compared with systemic administration, local delivery can maintain higher drug concentrations at the infection site while reducing systemic exposure and adverse effects [6]. Conventional local dosage forms, including solutions and suspensions, may be limited by local clearance and insufficiently sustained exposure [6,7]. Hydrogels provide hydrated polymer networks with tunable mechanics and release properties [7,8,9], supporting their investigation as local antibacterial delivery systems [10,11]. Hydrogels derived from natural polysaccharides such as chitosan may also exhibit intrinsic antibacterial activity, further expanding the design space [10,11]. Antibacterial hydrogel design must address not only the loading and release of bactericidal agents but also moisture maintenance, drainage, and tissue compatibility within wounds or cavities, requiring careful trade-offs among chemical composition, network structure, and biological function [12,13].

Among hydrogel platforms, injectable thermoresponsive hydrogels have attracted particular interest because of their operational convenience. These systems are flowable at room or lower temperature and form a gel in situ near physiological temperature after administration, which can allow them to conform to irregular infected cavities without additional crosslinkers or light exposure [14,15]. These properties motivate local delivery in osteomyelitic defects and periodontal pockets, although the relevant administration and infection endpoints differ between these sites [16,17,18]. In recent years, antibacterial components, tissue-repair factors, and multiresponsive elements have been integrated into thermoresponsive networks to create systems that combine in situ gelation, local retention, and anti-infective activity [19,20]. Thermally induced phase transitions can also be coupled with secondary triggers such as pH, enzymes, reactive oxygen species (ROS), or near-infrared (NIR) light to enable on-demand release and programmed treatment [3,21].

Recent reviews address thermoresponsive precision therapy [22], stimuli-responsive antibacterial dressings [23], localized biofilm treatment [3], intrinsically functional hydrogels [19], and photothermal infection control [21]. Di Martino et al. review injectable thermoresponsive local drug delivery, in vivo evidence, and translation [24]. The specific question addressed here is how formulation choices connect a deliverable thermoresponsive precursor to antibacterial exposure at a particular anatomical site. The analysis brings together three relationships: handling temperature and gelation kinetics govern placement; network and cargo architecture govern drug availability; and fluid turnover, tissue movement, and clearance govern residence. These relationships provide a basis for comparing design strategies and identifying the biological endpoints needed to evaluate them. Table 1 situates this focus within the complementary scopes of existing reviews.

This review follows a material–design–mechanism–application framework. Design sections describe how active components and release controls are assembled; mechanism sections examine what evidence connects those choices to bacterial killing or biofilm effects. Quantitative evidence is contextualized by the original assay and model, with separate attention to infected wounds, bone infection, periodontal disease, and ocular infection. Non-antibacterial barriers and other emerging delivery applications are addressed as future directions rather than established antibacterial indications.

Core evidence requires all three conditions: (1) delivery of a flowable formulation by needle, cannula, topical placement, or instillation before gelation; (2) a demonstrated temperature-triggered sol–gel transition near the intended tissue temperature; and (3) direct antibacterial or antibiofilm testing of the final formulation. The administration route is stated because an eye drop or topically applied gel does not establish needle injectability. Incorporation of an antibiotic, peptide, or metal does not substitute for testing the formulated product. Preformed thermally contracting gels, chemically crosslinked injectables without thermogelation, and systems supported only by an ingredient’s expected activity are contextual evidence. This review uses structured evidence mapping, without statistical pooling or cross-platform efficacy ranking.

**Table 1:** Descriptive positioning against overlapping review topics.

Existing Review Scope	Representative Sources	Contribution of This Review
General thermal and local-delivery platforms	Thermal precision therapy [22]; thermoresponsive local delivery [24].	Connects thermal handling and gelation to carrier-controlled exposure, residence, and antibacterial endpoints.
Stimuli-responsive antibacterial hydrogels	Stimulus-responsive dressings [23]; intrinsic hydrogel functions [19].	Separates thermal immobilization from infection-triggered release and antibacterial mechanism.
Biofilm-directed local delivery	Localized biofilm treatment [3].	Identifies planktonic, early-attachment, biomass, and established-biofilm endpoints separately.
Photothermal hydrogels	Photothermal infection control and repair [21].	Relates irradiation conditions, temperature exposure, and tissue safety to thermogel delivery.
Disease-specific local delivery	Osteomyelitis delivery [6].	Maps four anatomical settings to formulation requirements and meaningful endpoints.

### Literature Search and Evidence Assessment

A targeted literature search was completed on 11 September 2026 using PubMed (https://pubmed.ncbi.nlm.nih.gov/) and Europe PMC(https://europepmc.org/) publisher websites, and repositories. Searches combined thermoresponsive or thermosensitive hydrogel with antibacterial, antimicrobial peptide, biofilm, wound, osteomyelitis, periodontitis, or keratitis. Primary studies from 2020 to 2026 were prioritized, together with earlier publications on foundational mechanisms. The search supported a narrative synthesis and was not exhaustive; prospective registration and duplicate independent screening were not performed.

Relevance was assessed from titles and abstracts, followed by examination of full-text methods and results for administration, temperature-induced gelation, and activity of the complete formulation. Extracted information included composition, administration route, thermal behavior, release conditions, antibacterial endpoints, and animal models. Numerical results were interpreted with their original units, formulation, and experimental conditions. Core experimental evidence was distinguished from mechanistic background and non-antibacterial or non-thermogelling applications.

An author-defined A–D classification summarizes experimental coverage rather than risk of bias or clinical readiness. Levels A–C require demonstrated delivery, thermogelation, and direct in vitro antibacterial testing of the complete formulation. Level A additionally requires an established-biofilm assay, an infected-animal study with a post-treatment microbiological endpoint, and formulation-specific safety and retention assessment. Level B includes studies with a live-bacterial animal challenge; treatment of established infection and prophylaxis are specified separately. Level C comprises formulations evaluated in vitro, including studies with noninfected repair or ligature-induced periodontal disease models. The classification does not replace appraisal of the individual outcomes. Level D denotes activity inferred from ingredients or other reports and is excluded from the core comparison.

## 2. Fundamentals of Thermoresponsive Hydrogels

### 2.1. Thermoresponsive Mechanisms

Lower critical solution temperature (LCST), cloud point, and critical gelation temperature (CGT) describe different observations. The LCST belongs to a polymer–solvent phase diagram; a cloud point is an experimentally observed turbidity transition at a specified concentration and protocol. CGT is used here operationally for a macroscopic sol–gel transition, with the measurement method stated. A storage/loss-modulus crossover (G′ = G″) during a temperature sweep can indicate gelation, but its value depends on frequency, strain, heating rate, and history and is not a universal critical gel point. Frequency-dependent measurements and network connectivity provide complementary evidence [14,25,26,27,28,29]. Poly(N-isopropylacrylamide) (PNIPAM) commonly exhibits a transition near 32 °C in water, but phase separation or chain collapse does not by itself demonstrate injectable sol–gel conversion [26,28,30]. Poloxamers instead form associated micelles and, at an appropriate composition and concentration, a packed network. Figure 1 distinguishes these pathways; Table 2 separates phase-transition behavior from formulation-specific gelation [14,31,32].

Thermal phase behavior depends on polymer composition and the solution environment; macroscopic gelation additionally requires formulation-specific evaluation of network formation and co-loaded components [25,33]. In polysaccharide-based thermoresponsive materials, including chitosan/beta-glycerophosphate and methylcellulose, the transition temperature can be systematically adjusted through substitution degree, salt concentration, and pH [33,34]. In PNIPAM copolymers, the LCST can be tuned over a broad range by varying the ratio of hydrophilic to hydrophobic monomers [28,35]. Gelation kinetics are equally important: gelation that is too rapid may block the needle or prevent uniform filling, whereas gelation that is too slow can permit drug diffusion and loss. The practical gelation window is therefore a primary formulation-design parameter [20,29].

### 2.2. Representative Thermoresponsive Materials

Synthetic platforms offer different formulation and clearance trade-offs. For PNIPAM, residual monomer and synthesis impurities are toxicological questions distinct from the persistence of a largely nondegradable polymer backbone; purification does not solve long-term clearance [26,28,30,36]. Poloxamer 407 (Pluronic F127) is commonly used as the main thermogelling component, whereas poloxamer 188 (F68) often serves as a blend modifier; their gelation behavior is not interchangeable. Pharmaceutical use of a poloxamer does not establish approval or safety of every concentration, route, or composite formulation [31,37]. Dissolution or erosion of a water-soluble poloxamer gel should not be described as polymer biodegradation. Poly(lactic-co-glycolic acid)–poly(ethylene glycol)–poly(lactic-co-glycolic acid) (PLGA–PEG–PLGA) introduces hydrolytically degradable polyester segments, but degradation products and local pH remain formulation-specific concerns [15,38]. Methylcellulose derivatives can form networks through temperature-dependent association [34], and modified methylcellulose has also been investigated for vancomycin delivery [39].

Chitosan/β-glycerophosphate (β-GP) systems can undergo temperature-triggered gelation under suitable formulation conditions [10,33]. Unmodified chitosan gains cationic character through protonation of amino groups (–NH_2_ to –NH_3_^+^); it is not permanently positively charged. Antibacterial activity depends on pH, deacetylation, molecular weight, ionic strength, concentration, and contact time. Neutralization with β-GP can alter charge and activity, so the final gel requires direct testing. Quaternized derivatives have permanent cationic substituents and must be described separately [10,37]. Gelatin, hyaluronic acid, and cellulose-based systems also require formulation-specific modification or blending to obtain the intended thermal behavior. Natural origin, enzymatic susceptibility, or water solubility does not establish universal biocompatibility or complete in vivo degradation [10,31,33,34]. Table 2 summarizes these distinctions; Figure 2 links formulation choices to measurable delivery constraints.

**Table 2:** Material-level thermal behavior and formulation-specific evaluation requirements.

Material	Thermal Behavior/CGT	Administration and Safety Checks	Fate/Intrinsic Activity
PNIPAM and copolymers	PNIPAM commonly shows an aqueous phase transition near 32 °C; copolymer composition can shift it. CGT requires a separate formulation measurement.	Verify a pregel solution and injection force; quantify residual monomer separately.	Many backbones persist; purification does not confer degradability. No inherent antibacterial activity assumed.
Poloxamer 407/F127	Micellization and packing; CGT depends on concentration, salts, and cargo.	Frequently a principal gelling component; assess dilution and route-specific tolerance.	Dissolution/erosion is not biodegradation; measure clearance. Requires demonstrated activity or payload.
Poloxamer 188/F68	Often modifies a blend; not interchangeable with F127 as a thermogelling agent.	Report blend ratio and test final-formulation gelation.	Water solubility does not establish complete clearance or antibacterial activity.
PLGA–PEG–PLGA	Association-driven gelation depends on block structure and concentration.	Test handling window, acidic products, and local tissue response.	Polyester hydrolysis; quantify degradation and release separately. Payload-dependent activity.
Chitosan/β-GP	Temperature-induced association depends on pH, salt, and polymer characteristics.	Report molecular weight, deacetylation and pH; verify charge and activity after mixing.	Enzymatic breakdown is formulation dependent. Protonation is not permanent quaternization.
Modified methylcellulose	Association/clouding and macroscopic gelation require separate measurements.	Modification and additives affect thermal behavior; test drug-loaded formulation.	Mass loss does not prove backbone biodegradation. Requires antibacterial validation.
Gelatin/hyaluronic acid composites	Modification or blending must establish the intended warming-induced gelation.	Natural origin does not establish safety; test sterilized final product.	Enzyme sensitivity and clearance depend on chemistry; no universal antibacterial activity.

Note: CGT, critical gelation temperature; LCST, lower critical solution temperature; PNIPAM, poly(N-isopropylacrylamide); PLGA, poly(lactic-co-glycolic acid); PEG, poly(ethylene glycol); β-GP, β-glycerophosphate. CGT is a formulation- and method-dependent sol–gel transition. LCST/cloud point, dissolution, erosion, and chemical biodegradation are not interchangeable. Intrinsic antibacterial activity requires testing of the final unloaded formulation. The entries summarize design considerations rather than universal performance ratings [10,14,26,28,30,31,32,33,34,36,37,38,39].

### 2.3. Preparation and Crosslinking Strategies

The crosslinking and gelation mode of a thermoresponsive hydrogel directly affects administration. Physical networks may form through poloxamer micelle packing, hydrophobic association, hydrogen bonding, or host–guest interactions. Formulations intended for local thermal gelation must remain flowable during administration and form a gel near the target-tissue temperature. Low-temperature flowability and shear-thinning are distinct properties; not all injectable thermoresponsive systems rely on shear-thinning [20,28]. Chemically crosslinked systems, including photocrosslinked, enzyme-crosslinked, and click-chemistry systems, can provide more stable mechanical properties. Their inclusion as core injectable thermogelling formulations additionally requires a demonstrated temperature-triggered sol–gel transition near the intended tissue temperature. Double networks, nanocomposites, and dynamic covalent bonds can improve toughness or prolong retention, but may alter injection force, gelation kinetics, and degradation. These properties should therefore be evaluated together within the same formulation [9,20,40,41].

Preparation routes can be grouped into three categories: direct gelation of a thermoresponsive polymer solution; incorporation of drug-loaded nanoparticles or microspheres into a thermoresponsive matrix; and combination of a thermoresponsive network with a second controllable crosslinking mechanism. Direct gelation is operationally simple and suitable for immediate local administration, but hydrophilic small molecules may undergo rapid initial diffusion. Pre-encapsulating drugs in nanoparticles or microspheres before dispersing them in a thermoresponsive matrix may reduce burst release and extend delivery [16]. Porous nanofillers such as metal–organic frameworks (MOFs) may also provide drug-binding sites or metal nodes, but improvements in antibacterial activity, release, or mechanics must be demonstrated separately using formulation-specific controls and cannot be inferred solely from the general properties of MOFs [42].

### 2.4. Key Properties and Characterization

Characterization should establish administration, gel formation, post-gel mechanics, and the release–residence relationship. Report needle or cannula dimensions, temperature, flow rate, and injection force for injected products; viscosity and drop delivery are more relevant to instilled formulations. Tube inversion, turbidity, calorimetry, and rheology probe different events and should not be treated as interchangeable gel-point methods. For a rheological CGT, report the crossover criterion, oscillation frequency, strain within the linear viscoelastic range, temperature ramp, and replicate variability. Frequency and strain sweeps test network behavior beyond a single crossover [15,20,29]. Swelling describes water uptake by the polymer network [43] and must be distinguished from mass loss, molecular degradation, and disappearance from the administration site: loss of gel mass alone does not demonstrate clearance of polymer or nanofillers [7,40].

Tissue dilution, salts, proteins, motion, and fluid turnover can change gelation, release, and adhesion. Thermal gelation can improve localization, but cannot guarantee retention. In vivo imaging should account for whether the signal follows intact material or a released label, and should be complemented by drug or material measurements where possible [20,44]. Shelf stability, sterilization, mixing reproducibility, and the permitted handling interval before administration are additional formulation attributes [29,45].

## 3. Antibacterial Design Strategies

### 3.1. Antibiotic Loading and Controlled Release

Antibiotic loading provides a direct route to thermoresponsive local treatment. Tao et al. assembled vancomycin-containing nanoparticles from quaternized and carboxylated chitosan and dispersed them in a chitosan/α,β-glycerophosphate thermogel. The reported encapsulation efficiency and drug loading were (60.1 ± 2.1)% and (24.1 ± 0.84)%, respectively, with in vitro release extending to 26 days; the study also used a rabbit osteomyelitis model [16]. Yuan et al. investigated vancomycin in a PLGA–PEG–PLGA/hydroxyapatite system [38]. Zhi et al. evaluated vancomycin-loaded butyl-glycidyl-ether-modified methylcellulose that gelled in approximately 10 min at 37 °C and released vancomycin over 28 days in vitro. Following debridement in a rat model of *S. aureus*-induced osteomyelitis, local injection of the formulation reduced the cultivable bacterial burden in tibial tissue at the four-week endpoint [39]. Table 3, Table 4 and Table 5 summarizes representative formulations and their assay context.

Carrier architecture can also separate antibacterial delivery from regenerative signaling. Kai et al. incorporated teicoplanin and Mg/Zn-doped β-tricalcium phosphate into a poloxamer system [46]. In the formulation of Zheng et al., vancomycin was loaded in the thermoresponsive polypeptide gel, whereas the tetrapeptide Asp–Gly–Glu–Ala (DGEA) was loaded in PLGA microspheres [17]. The intended sequence is earlier antibiotic exposure followed by prolonged peptide delivery. Differences in payload, dose, formulation, and model prevent direct efficacy ranking.

**Table 3:** Representative thermoresponsive formulations, delivery characteristics, and biological evaluation.

System/Reference	Thermal Behavior/Delivery	Loading, Release and Residence	Evidence Level and Biological Evaluation
VCM-NPs in CS/α,β-GP [16]	Sol at 20 °C; gel at 37 °C; syringe delivery and rheological characterization	EE 60.1 ± 2.1%; DL 24.1 ± 0.84%; ~65% release at 26 d in PBS, 37 °C	B; in vitro *S. aureus* inhibition and rabbit osteomyelitis model
Gel/VA-MP/DGEA [17]	8 wt% bare gel transitions at ~29 °C; microspheres lower transition; local injection	VA in gel; DGEA in PLGA microspheres; sequential release	B; in vitro *S. aureus*/*E. coli* inhibition and rat osteomyelitis model
Emodin/P188/F127/CMC [47]	E1/E2 gel at 34/33 °C; extrusion through a 23-gauge needle at 24 °C	64% released within 24 h; release monitored to 96 h	C; in vitro *P. gingivalis* inhibition; bone and inflammatory outcomes in ligature-induced rat periodontitis
CFMD: CP/FA-MBG/doxycycline [18]	20 wt% CP matrix: transition onset ~27 °C, fully gelled by 34 °C; extrusion through a 25-gauge needle.	Doxycycline-loaded FA-MBG nanoparticles dispersed in CP hydrogel	C; in vitro bacterial and preformed-biofilm assays; rat ligature/LPS periodontitis model
Chitosan/hyaluronic acid, levofloxacin + betamethasone [48]	Unloaded Ch–HA–35 matrix: ~8 min at 37 °C, prepared using 35% *w*/*v* β-GP stock; drug-loaded formulation delivered by instillation.	24 h: levofloxacin 49.6 ± 2.1%, betamethasone 30.3 ± 1.8%	C; in vitro antibacterial assays and noninfected rabbit corneal-injury model
Erythromycin/BPEP [49]	Unloaded 0.5/1/2BPEP matrices: CGT 32/28/24 °C at 10/8/6 wt%, respectively; erythromycin formulation administered by instillation.	Visible ocular gel persisted after blinking	B; in vitro *S. aureus* inhibition and bacterial keratitis model in mice
FQT1/8: F127/QCS/TA [50]	16/1.25/0.125% *w*/*v*; liquid below 25 °C, gel at 35 °C; instillation	Fluorescent tracer remained visible at 8 h after 100 μL topical administration	B; in vitro *S. aureus* assays and perioperative infection prophylaxis in rabbits
PDA/PCLGA–PEG–PCLGA@Ag [51]	30 wt% matrix crossover near 34 °C; Ag-loaded solution injected into infected wounds; gel within 10 min	~40% cumulative total Ag release at 96 h, measured by ICP	B; in vitro *E. coli*/*S. aureus* assays and *S. aureus*-infected rat wound model

Note: VA/VCM, vancomycin; NPs/MPs, nanoparticles/microspheres; CS, chitosan; GP, glycerophosphate; CMC, carboxymethylcellulose; CP, covalent chitosan-F127 conjugate; FA-MBG, folate-modified mesoporous bioactive glass; CFMD, doxycycline-containing CP/FA-MBG formulation; QCS, quaternized chitosan; TA, tannic acid; P188, poloxamer 188; F127, poloxamer 407; DGEA, Asp-Gly-Glu-Ala; PDA, polydopamine; PCLGA, poly(ε-caprolactone-co-glycolide); PEG, poly(ethylene glycol). Ch–HA, chitosan-hyaluronic acid; PLGA, poly(lactic-co-glycolic acid); EE, encapsulation efficiency; DL, drug loading; PBS, phosphate-buffered saline; CGT, critical gelation temperature; ICP, inductively coupled plasma; LPS, lipopolysaccharide. E1 and E2 denote the emodin-containing formulations reported in Ref. [47]. BPEP denotes poly(BPOSS/PEG/PPG urethane), where BPOSS is bifunctional polyhedral oligomeric silsesquioxane and PPG is poly(propylene glycol). FQT denotes F127/QCS/TA; FQT1/8 is the formulation label used in Ref. [50]. Organisms: *S. aureus*, *Staphylococcus aureus*; *E. coli*, *Escherichia coli*; *P. gingivalis*, *Porphyromonas gingivalis*.

**Table 4:** Bacteriological and animal-model context.

System/Reference	Bacteriological and Animal Model
VCM-NPs in CS/α,β-GP [16]	*S. aureus* assays; rabbit tibial inoculation; infection-related inflammation and bone repair
Gel/VA-MP/DGEA [17]	*S. aureus*/*E. coli* assays; inoculated rat femoral model with debridement
Emodin/P188/F127/CMC [47]	Free-emodin MIC = MBC 50 μM against *P. gingivalis*; ligature-rat bone/inflammation outcomes
CFMD: CP/FA-MBG/doxycycline [18]	97.6%/96.9% colony reduction for *P. gingivalis*/*S. mutans*; preformed 3-d biofilms treated 48 h; ligature + LPS rat model
Chitosan/hyaluronic acid, levofloxacin + betamethasone [48]	Zones: *S. aureus* 17.8 ± 1.5 mm, *E. coli* 14.2 ± 1.8 mm, *S. epidermidis* 11.9 ± 0.8 mm; noninfected rabbit corneal ethanol-burn model
Erythromycin/BPEP [49]	*S. aureus* inhibition; bacterial-inoculated mouse keratitis; repeated ocular safety
FQT1/8: F127/QCS/TA [50]	*S. aureus* co-culture; rabbit perioperative live-bacterial challenge and aqueous-humor culture
PDA/PCLGA–PEG–PCLGA@Ag [51]	*E. coli*/*S. aureus* spread-plate testing; *S. aureus*-inoculated rat wounds with post-treatment wound-swab cultures; host ROS scavenging evaluated separately

Note: MIC, minimum inhibitory concentration; MBC, minimum bactericidal concentration; ROS, reactive oxygen species. Organisms: *S.mutans*, *Streptococcus mutans*; *S. epidermidis*, *Staphylococcus epidermidis*.

**Table 5:** Antibacterial assay conditions, controls, and release and material evaluation.

System/Reference	Inoculum, Medium and Exposure	Key Comparators and Endpoint	Release and Material Evaluation
VCM-NPs in CS/α,β-GP [16]	*S. aureus* cultured in LB to OD_600_ = 0.5. Bacteria exposed to gel extracts overnight at 37 °C; agar diffusion: 24 h, with daily transfer in prolonged testing.	PBS, unloaded gel, free VCM, VCM-NPs and VCM/gel; OD_600_ and inhibition zones. Intracellular-infection imaging was also performed.	Dry-mass loss in 0.5 mg/mL lysozyme at 37 °C and 100 rpm; VCM release in PBS at 37 °C for 26 d.
Gel/VA-MP/DGEA [17]	*S. aureus*/*E. coli* in LB; turbidity assay initial OD_625_ ≈0.05, 37 °C, 100 rpm, 2–24 h. Agar: 100 µL inoculum, 6 mm/2 mg discs; 24 h, 3 and 7 d.	Bacteria-only, Gel/MP and vancomycin-containing gel comparators; optical density and diffusion zones. Free VA and DGEA groups were included in the animal study.	In vitro mass-loss testing in PBS with or without 2 mg/mL elastase, 37 °C, 70 rpm; subcutaneous observation to 42 d used 8 wt% polymer/5 wt% microspheres.
Emodin/P188/F127/CMC [47]	*P. gingivalis* ATCC 53978, OD_600_ 0.01, blood agar, anaerobic 48 h; 15 µL gel/disc. Free-emodin MIC: OD_600_ 0.1 in supplemented BHI, 48 h; MBC plating: 7 d.	Vehicle gel, PBS and 0.12% chlorhexidine; inhibition zones with 15 µL gel or 5 µL chlorhexidine per disc. Free-emodin MIC/MBC were measured separately.	Release from 200 µL gel into 1 mL PBS at 37 °C and 50 rpm, monitored to 96 h; emodin quantified by absorbance at 260 nm.
CFMD: CP/FA-MBG/doxycycline [18]	*P. gingivalis*/*S. mutans*: 100 µL of 1.5 × 10^8^ CFU/mL +1 mL gel +1 mL BHI, anaerobic 37 °C/24 h; blood-agar recovery 5 d. Biofilms: 3 d preformation, 48 h gel exposure.	Untreated, CP and CFM controls; colony counts, live/dead imaging and biofilm thickness.	Subcutaneous CP matrix showed 76.1% mass loss at 4 weeks.
Chitosan/hyaluronic acid, levofloxacin + betamethasone [48]	*E. coli* ATCC 25922, *S. aureus* 25923, *S. epidermidis* 12228; 0.5 McFarland (≈1–2 × 10^8^ CFU/mL), 100 µL on Mueller–Hinton agar; 6 mm gel discs, 37 °C/24 h.	Unloaded Ch–HA and 5 µg levofloxacin discs; single- and dual-drug formulations; inhibition-zone diameters.	Ch–HA-35 mass-loss testing in PBS, pH 7.4, at 37 °C for 30 d; visible ocular persistence for 6–8 h after instillation.
Erythromycin/BPEP [49]	*S. aureus*, 1 × 10^8^ CFU/mL on tryptone-soy agar; 6 mm discs, 5 µL solution, 24 h. Animal challenge: 2 µL of 5 × 10^7^ CFU/mL intrastromally; eye drops began 24 h later.	PBS, unloaded BPEP/F127, free erythromycin and F127–erythromycin; inhibition zones. Animal outcomes: clinical score, imaging and inflammation.	PBS mass-loss assay at 34.5 °C and 50 rpm; the 2BPEP gel persisted for >6 d in vitro. Ocular gel persistence was assessed by visual observation.
FQT1/8: F127/QCS/TA [50]	*S. aureus*, 1 × 10^8^ CFU/mL mixed 1:1 with gel, 37 °C/2 h; LB-agar recovery and OD_600_. Diffusion-zone assay: 24 h.	Saline, levofloxacin and TA-free FQ; colonies, OD_600_ and diffusion zones. Perioperative dosing began before bacterial challenge; aqueous-humor cultures assessed infection prophylaxis.	In vitro erosion assay in PBS at 35 °C and 100 rpm; ocular fluorescent-tracer imaging through 8 h.
PDA/PCLGA–PEG–PCLGA@Ag [51]	250 µL precursor (30 wt%, 500 µg/mL AgNPs) was gelled at 37 °C for 10 min before adding 250 µL *E. coli*/*S. aureus* at 10^6^ CFU/mL; exposure at 37 °C for 24 h.	P2 and PDA/P2–4 controls; spread plates. Rat wounds received 200 µL *S. aureus* at 10^9^ CFU/mL, followed by treatment after 24 h and wound-swab cultures on days 5 and 15.	Cumulative total Ag release was measured by ICP over 96 h.

Note: CFM, CP/FA-MBG formulation; FQ, F127/QCS; AgNPs, silver nanoparticles; LB, lysogeny broth; BHI, brain heart infusion; OD, optical density, with the subscript indicating the measurement wavelength in nm; CFU, colony-forming units; ATCC, American Type Culture Collection. P2 denotes the PCLGA–PEG–PCLGA copolymer formulation in Ref. [51], and PDA/P2–4 denotes its PDA-modified formulation.

Carrier architecture determines which release processes can be controlled independently. In Tao et al., nanoparticle incorporation reduced the initial vancomycin burst relative to free nanoparticles or directly loaded gel [16]. The gel–microsphere design of Zheng et al. instead separated antibiotic and regenerative-peptide delivery [17]. These examples support compartmentalization as a design strategy, while direct loading into polyester or modified-methylcellulose matrices provides alternative routes to prolonged release [38,39]. Release duration alone cannot rank these architectures: release medium, sink conditions, dialysis barriers, sample replacement, dose recovery, and drug stability affect the measured profile. Percentage release describes mass transfer rather than the active concentration at an infection site; matrix mass loss likewise does not establish molecular degradation or systemic clearance. Formulation selection therefore depends on the required onset and duration of antibacterial exposure, balanced against local tissue tolerance and material residence. MIC, MBC, and biofilm susceptibility represent different assays and must be interpreted for the relevant pathogen and treatment context [2,5,6,52].

### 3.2. Inorganic Antibacterial Nanomaterials

Nanomaterial design is organized by carrier structure rather than by assuming a common antibacterial mechanism. Metal and metal-oxide particles can release ions and interact with bacterial envelopes; two-dimensional layered double hydroxides (LDHs) and MXenes add surface-area and dispersion considerations; porous metal–organic frameworks (MOFs) offer cargo-binding and transport pathways; and nanozymes are defined by measured catalytic activity. These categories can overlap. Composition and oxidation state, size and shape, surface charge, aggregation in physiological media, and ion-release rate affect bacterial exposure and host toxicity [42,53,54,55,56,57]. General antibacterial nanomaterial reports establish mechanistic context, but only final formulations meeting the delivery, thermogelation, and antibacterial criteria enter the core evidence map. For MXene- and MOF-containing systems, these properties require direct validation in the complete formulation.

For metal-containing systems, ion release should be measured separately from particle leakage. A particle-free ion control tests whether activity is explained by released species, while a chelator or ion-depletion experiment can support the mechanism only if its independent bacterial effects are controlled [53,56]. Essential elements such as Mg, Zn, and Cu can still be toxic at high local exposure. Metal tolerance may involve efflux, sequestration, biofilm protection, or other adaptive responses; independence from a conventional drug target does not imply negligible resistance risk [53,56]. The Cu-LDH/F127 eye-drop system of Ikwuagwu et al. combined copper-containing nanosheets with in situ thermal gelation. In a mouse *S. aureus* keratitis model, a five-day course of daily topical Cu-LDH@Gel with 808 nm irradiation at 1 W cm^−2^ for 10 min reduced recovered colony counts relative to Cu-LDH@Gel without irradiation and Cu-LDH nanosheets with or without irradiation. The corneal temperature reached approximately 43 °C under this protocol, linking the antibacterial outcome to a defined thermal exposure [58].

Ge et al. investigated a nanocomposite based on the selected poly(ε-caprolactone-co-glycolide)–PEG–poly(ε-caprolactone-co-glycolide) (PCLGA–PEG–PCLGA) copolymer (P2) and its polydopamine (PDA)-modified formulation (PDA/P2–4). At 30 wt%, the PDA/P2–4 matrix showed rheological crossover near 34 °C; its silver nanoparticle (AgNP)-loaded solution was injected into *S. aureus*-infected rat wounds and formed a gel within 10 min. Antibacterial testing distinguished the Ag-containing gel from the polymer and PDA-modified controls, while ROS scavenging was assessed separately in host-cell assays. Approximately 40% cumulative total silver release was measured by 96 h using inductively coupled plasma (ICP) analysis. The formulation combined temperature-triggered local delivery with antibacterial and antioxidant functions [51].

### 3.3. Antimicrobial Peptides and Natural Bioactive Compounds

Antimicrobial peptides (AMPs) differ in sequence, charge, hydrophobicity, secondary structure, and target. Membrane permeabilization and depolarization are common mechanisms, but some peptides act on intracellular targets or host signaling [59,60,61,62]. Physical encapsulation can protect a peptide yet restrict diffusion; covalent tethering can improve retention yet impair the conformation or accessibility needed for killing. Formulation evaluation should compare free peptide, blank gel, and peptide-loaded gel, measure activity after gelation and during release, and assess proteolysis, salt sensitivity, protein binding, hemolysis, and mammalian-cell toxicity. Peptide resistance and biofilm tolerance remain possible [61,62,63]. Specific thermoresponsive examples are discussed separately from general peptide-hydrogel design.

Feng et al. linked the self-assembling RADA16 segment to the antibacterial sequence RRWRVIVKW and incorporated the conjugate into PNIPAM; an MGF E (mechano-growth-factor E) peptide was added separately as a regenerative cargo. The resulting formulation exhibited temperature-dependent gel formation and was evaluated for wound repair in noninoculated full-thickness rat skin defects. Antibacterial assays tested the Amps and RA-Amps peptides against *Escherichia coli* and *S. aureus*. This study illustrates the integration of an antibacterial peptide design with a thermoresponsive matrix and a separate regenerative cargo [64].

Ma et al. incorporated the histatin-derived peptide Nal-P-113 and antioxidant polydopamine nanoparticles into a chitosan/β-GP thermogel for periodontal delivery. Gel formation was observed at 37 °C. Single-species colony-count assays and mixed-inoculum viability and adhesion imaging supported activity against *Streptococcus gordonii*, *Fusobacterium nucleatum*, and *Porphyromonas gingivalis*. Repeated delivery of the pregel into periodontal pockets reduced alveolar bone loss and inflammatory tissue changes in rats with ligature-induced periodontitis [65]. These findings support local delivery and periodontal tissue protection.

Natural bioactive small molecules form a distinct category from AMPs and cationic polysaccharides. Shim et al. incorporated emodin into a poloxamer/carboxymethylcellulose thermogel and examined antibacterial activity, release, and periodontal tissue outcomes. Free emodin had an MIC and MBC of 50 μM against *P. gingivalis* [47]. Li et al. demonstrated that berberine co-assembled with baicalin into nanoparticles with greater antibacterial activity than free berberine in the reported *S. aureus* assays, whereas co-assembly with wogonoside produced nanofibers with lower activity than free berberine. These co-assembled nanostructures illustrate composition- and morphology-dependent antibacterial activity and provide design context for natural-compound delivery [66]. Solubility, chemical stability, batch identity, retained activity, and local dose must be established for each natural compound. Unmodified chitosan and permanently cationic quaternized derivatives are distinguished as described in Section 2.2 [10,37].

### 3.4. Contact-Killing and Antifouling Surfaces

Contact-active designs immobilize membrane-interacting groups in the network; antifouling designs reduce initial adhesion through interfacial hydration or other surface properties. These are different objectives. Chen et al. compared drug-free poloxamer/quaternized-chitosan gel, levofloxacin solution, and the drug-loaded gel. The drug-free formulation showed partial antibacterial activity, but these comparisons did not isolate nonleaching contact killing from effects of soluble cationic components [37]. Molecular docking or simulation can motivate a binding hypothesis but does not establish bacterial killing, membrane penetration, or an in vivo mechanism. Comparing drug-free gel, free drug, leachate, and immobilized versus soluble cationic components is necessary to distinguish contributions. Non-thermogelling antifouling coatings remain adjacent design evidence [3,10,37].

The density and accessibility of fixed charges, chain mobility, surface hydration, and protein fouling should be considered together with mammalian-cell compatibility. An antifouling surface may prevent early colonization without killing bacteria, whereas a contact-active surface may fail to reach organisms deep in a mature biofilm. Claims of nonleaching action require chemical analysis of the released fraction and testing of that fraction at relevant exposure times [3,10,37].

### 3.5. Release Triggers and Combined Treatment

Thermal gelation is the initial delivery and immobilization step. A second stimulus can regulate release or activate an antibacterial component, but the stimulus and mechanism should be named separately. Near-infrared (NIR) irradiation is an input; photothermal conversion produces heat; photodynamic treatment generates reactive species through a photosensitizer. pH-, enzyme-, and ROS-responsive linkers instead regulate activation or transport. Liu et al. incorporated PDA into a cold-prepared chitosan/β-GP mixture that formed gels at 37 °C and demonstrated NIR-assisted reductions in culturable *S. aureus* and *E. coli*, providing an example of photothermal integration [67]. Huang et al. prepared a covalently crosslinked PNIPAM-based network with temperature-dependent phase behavior, pH-responsive swelling, and NIR-assisted antibacterial activity; this preformed network provides mechanistic context for responsive wound dressings [68]. Each additional component can alter the CGT, drug partitioning, and tissue exposure, so these attributes should be measured in the final composite.

Combined treatment should be evaluated with a factorial set of material, drug, and stimulus controls; an enhanced endpoint alone does not establish synergy. The thermogel containing doxycycline-loaded, folate-modified mesoporous bioactive glass reported by Yu et al. links cargo delivery to antibacterial and periodontal-repair outcomes [18]. Infection-associated cues may provide further control, but their selectivity requires a nonresponsive formulation and physiologically relevant cue concentrations. ROS generation intended for bacterial killing and ROS scavenging intended to protect host tissue are distinct functions whose spatial and temporal compatibility must be tested [18,19,21,69]. Figure 3 separates thermal localization, secondary activation, and the resulting mechanism.

## 4. Antibacterial Mechanisms

### 4.1. Thermoresponsive Network Confinement and Contact Killing

Temperature-triggered network formation can change diffusion and residence, but these delivery effects are not bactericidal mechanisms in themselves [14,20]. A retained drug can kill through its molecular target; an accessible immobilized cationic group may interact with the bacterial envelope. In the poloxamer/quaternized-chitosan/levofloxacin system [37], a mechanistic assignment requires separating free-drug activity, soluble cationic material, and the gel surface. Comparing sol and gel states is informative only if dose, exposure time, and temperature effects on bacterial growth are controlled. The core mechanistic controls and readouts are summarized in Table 6.

### 4.2. Photothermal and ROS-Mediated Killing

Photothermal conversion can raise local temperature after irradiation, whereas photodynamic or catalytic pathways can generate ROS. Neither pathway is established by observing NIR exposure alone. Interpretation requires reporting wavelength, power density at the sample, exposure duration, geometry, and temperature–time history; tissue depth, thermal nonuniformity, and collateral damage limit interpretation [21,67]. In vitro, irradiation of the PDA/chitosan/β-GP formulation at 808 nm and 0.5 W cm^−2^ for 10 min reduced recovered colony counts by 91.22% for *S. aureus* and 96.69% for *E. coli* relative to the no-hydrogel control [67]. An externally heated control matched for temperature–time exposure helps distinguish heat from chemical or photochemical activity. Fluorescent ROS measurements require caution because some organic dyes lack specificity for individual ROS [69]. For composite hydrogels, probe–material interference and the effects of light or heating on the readout should also be tested, with scavenger-only controls when scavengers are used. Gas-mediated or non-thermogelling antibacterial platforms provide mechanistic context; their activity cannot be extrapolated to an injectable thermogel without validation of the complete formulation.

### 4.3. Thermoresponsive Sustained Release and Antibiofilm Mechanisms

Biofilm-associated tolerance can arise from drug-dependent transport and matrix interactions, slow growth, metabolic heterogeneity, and persister subpopulations [4,70,71,72]. Sustained release from a thermogel may maintain exposure, but does not by itself prove penetration or eradication of an established biofilm. Preventing attachment, reducing total biomass, disrupting extracellular polymeric substances (EPS), and reducing viable biofilm bacteria are separate outcomes [3,73]. Crystal violet staining measures attached biomass, while viable counts after validated biofilm recovery measure culturable organisms. Live/dead imaging and EPS visualization add spatial information but do not replace viable counting. Mature-biofilm claims should state strain, biofilm age, substrate, medium, exposure, recovery method, and detection limit. Minimum biofilm eradication concentration (MBEC) requires a defined biofilm assay and should not be inferred from a planktonic MIC.

### 4.4. Immunomodulatory and Pro-Regenerative Mechanisms

Antibacterial, immune, and regenerative endpoints can improve concurrently without establishing their causal order. Yu et al. reported antibacterial activity together with macrophage-phenotype and periodontal-bone changes [18]. Evidence for osteogenesis should identify the actual endpoint, such as alkaline phosphatase (ALP), runt-related transcription factor 2 (RUNX2), osteocalcin (OCN), osteopontin (OPN), mineral deposition, microcomputed tomography, or histomorphometry; these measurements are not interchangeable. Macrophage markers and cytokines characterize a phenotype rather than prove that an M2-like state caused regeneration. Causal studies require appropriate pathway inhibition, depletion, or other perturbation alongside blank-gel and free-drug controls. Tissue repair and infection control should therefore be reported as separate endpoint domains [74,75,76,77].

### 4.5. Methods for Mechanistic Evaluation

Mechanistic testing should connect formulation state, delivered exposure, bacterial outcome, and host response. Colony-forming units (CFU), optical density (OD), inhibition-zone diameter, biomass, and fluorescence viability are different measurements. For viable counts, percentage reduction is 100 × (N_control_ − N_treatment_)/N_control_, and log_10_ reduction is log_10_(N_control_/N_treatment_), where the control and treatment use the same sampling and recovery method. Conversion from a percentage to a log reduction is valid only for the same positive count-based endpoint; OD, inhibition zones, and biomass percentages cannot be converted to CFU reduction. Zero colonies indicate a result below a method-specific detection limit, not proof of sterility. Table 3, Table 4 and Table 5 preserves assay context, and Table 6 maps causal claims to the minimum controls and complementary readouts [3,11,63,73]. Figure 4 summarizes the separate evidence pathways for bacterial killing, biofilm effects, and host repair.

## 5. Biomedical Applications

### 5.1. Infected Wound Healing

Chronic wounds impose a substantial clinical burden, particularly in diabetes [78,79,80]. Tissue repair involves coordinated inflammatory, cellular, and extracellular-matrix responses [75,76,77]. Infected wounds additionally require control of bacterial burden alongside restoration of tissue integrity. Colonization, clinically infected wounds, and deliberately inoculated animal models should be distinguished. A thermogel may conform to a defect and sustain local exposure, but successful use also depends on fluid handling, adhesion, oxygen transport, atraumatic removal, and compatibility with dressing changes [11,21]. For photothermal designs, illumination depth and temperature uniformity are additional constraints. Table 7 maps these requirements to meaningful preclinical endpoints rather than assigning one universal modulus or gelation temperature to all wounds. Figure 5 illustrates the anatomical constraints across the four application areas.

The wound studies illustrate distinct ways in which temperature affects treatment. Ge et al. injected an Ag-containing thermoresponsive precursor into infected rat wounds, where it formed a gel; treatment reduced bacteria recovered from wound swabs [51]. Liu et al. combined a PDA/chitosan/β-GP formulation that gelled at 37 °C with topical NIR treatment and observed decreased bacterial recovery after the first treatment of *S. aureus*-infected wounds [67]. Preformed PNIPAM networks offer a different mechanism: warming changes contraction or transport within an existing gel. Gou et al. studied antibacterial activity in vitro and repair in noninoculated wounds [81]; Gong et al. demonstrated syringe extrusion, temperature-dependent release, and reduced colony recovery from infected wounds [82]; Xu et al. combined thermal contraction with photodynamic treatment and reported antibiofilm effects and reduced culture-based bacterial burden in infected diabetic wounds [83]. Irani et al. examined F127/polyglutamic acid sol–gel behavior and extrusion, followed by topical treatment and healing assessment in an infected-wound model [84]. For cavity filling, the relevant thermal function is formation of a depot after placement; for a preformed surface dressing, contraction and transport can contribute separately. This distinction also guides outcome interpretation: wound closure integrates mechanical contraction and host repair, whereas recovered viable bacteria assess antibacterial effects [11,75,76].

### 5.2. Bone and Joint Infections

Bone-infection designs combine local antibacterial delivery with support for subsequent defect repair. Vancomycin-containing chitosan nanoparticle gels and modified methylcellulose matrices address local antibiotic delivery [16,39]. Yuan et al. incorporated hydroxyapatite into a vancomycin-loaded PLGA–PEG–PLGA formulation [38], while Kai et al. combined teicoplanin with Mg/Zn-doped β-tricalcium phosphate in a poloxamer gel [46]. These mineral-containing composites modify the local carrier environment. Zheng et al. instead separated vancomycin in the gel from the osteogenic peptide DGEA (Asp–Gly–Glu–Ala) in PLGA microspheres to investigate sequential delivery [17]. The studies thus address complementary aspects of bone repair: the carrier environment and the timing of antibacterial and regenerative signals. Evaluating either approach requires bacterial control to be assessed alongside bone formation; greater mineralization alone cannot establish resolution of infection.

Infected bone defects require dead-space filling, suitable injected volume, persistence under local compression, and integration with debridement and systemic therapy [6,52,85,86]. Hydrogels can conform to a cavity and accommodate sequential delivery but ordinarily provide less structural support than a load-bearing cement. Nondegradable polymethyl methacrylate (PMMA) carriers have different mechanical, elution, and removal considerations; a hydrogel is not automatically a replacement for fixation or cement. Release duration should not be equated with the required clinical treatment course. Relevant endpoints include quantitative bone or implant cultures, recurrence after treatment, microcomputed tomography, histology, and material/drug residence. Implant coatings are a different administration format from injectable cavity fillers [6,85,86,87].

### 5.3. Periodontal and Oral Infections

Periodontitis combines a dysbiotic biofilm with host-mediated destruction of supporting tissues [88,89]. A local thermogel must therefore accommodate both microbial control and a changing inflammatory environment while remaining deliverable into a pocket exposed to saliva, crevicular flow, and chewing. Yu et al. combined doxycycline-loaded mesoporous bioactive glass with a thermogel, distributing antibacterial and regenerative functions across a particle–matrix composite [18]. Shim et al. incorporated emodin into a poloxamer/carboxymethylcellulose formulation and examined antibacterial activity together with inflammatory and alveolar-bone outcomes [47]. These examples connect local delivery to host-tissue preservation through different compositions. The ligature/LPS model used by Yu et al. and the ligature model used by Shim et al. primarily inform periodontal inflammation and supporting-tissue responses. Demonstrating that prolonged delivery drives microbial control requires the additional connection between pocket residence, local drug concentrations, and biofilm viability. Consequently, in vitro release and improvement in bone endpoints represent complementary parts of the delivery hypothesis, not interchangeable measures of pocket-level efficacy.

### 5.4. Ocular Infections

Topical ocular delivery is constrained by rapid lacrimal clearance and corneal and conjunctival epithelial barriers [90]. Thermal gelation addresses residence after instillation, but its practical benefit depends on balancing retention with optical clarity, tolerable viscosity, pH, osmolality, and epithelial compatibility. Chen et al. investigated a poloxamer/quaternized-chitosan levofloxacin formulation [37]. Aghamirsalim et al. combined levofloxacin and betamethasone in chitosan–hyaluronic acid and evaluated repair in a noninfected chemical corneal-injury model [48]. The erythromycin/polyhedral oligomeric silsesquioxane (POSS) hybrid and Cu-LDH/F127 eye drops were directed at bacterial keratitis [49,58], whereas the F127-based perioperative formulation was administered prophylactically around cataract surgery [50]. These studies address established infection, infection prevention, and injury repair as separate therapeutic settings. Ocular formulation selection should pair the relevant bacterial endpoint with residence and epithelial recovery; prolonged visible or fluorescent retention alone does not establish sustained therapeutic drug exposure. Instillation performance also remains distinct from needle injectability, and a steroid-containing repair formulation cannot be extrapolated to uncontrolled infection.

## 6. Current Challenges

Manufacturing and material fate constrain the same design choices used to improve delivery. Residual N-isopropylacrylamide and persistence of polymerized PNIPAM require separate control strategies [28,30,36]. Double-network reinforcement can improve toughness [41], but the resulting formulation must retain an appropriate injection force and gelation window [29]. Similarly, adding carrier particles or multiple active components expands the number of composition and stability attributes that must be controlled. Sterilization and storage therefore need to preserve formulation performance, including handling behavior, gelation, release, and biological activity. Critical quality attributes include polymer identity and composition, residual impurities, drug content, particle size where applicable, rheology at handling and tissue temperatures, release reproducibility, sterility, endotoxin burden, and stability during the intended handling interval [20,29,45]. A formulation that performs well immediately after preparation is translationally useful only if these linked attributes remain reproducible throughout manufacture and use.

Biological risks include metal or particle retention, oxidative injury, peptide hemolysis or instability, and prolonged subinhibitory antibiotic exposure [3,53,56,62]. Cell viability over a short incubation does not establish tissue safety or clearance. Animal studies should identify inoculation or disease induction, strain, dose, group size, randomization, blinding, follow-up, and adverse findings. Current evidence is predominantly preclinical. Differences in formulation composition, assay conditions, and animal models limit cross-study comparison and motivate standardized evaluation of antibacterial exposure, tissue response, and long-term material fate.

Clinical positioning should specify whether the formulation is intended for local drug delivery, a barrier, a dressing, or another purpose and should be assessed within the applicable jurisdiction. Prior use of a polymer excipient does not validate a new product or route [45,91]. Local hydrogel delivery must be integrated with debridement, implant management, and systemic treatment where appropriate [6,85]. Translational claims require formulation-specific manufacture, safety, pharmacokinetic, and clinical evidence rather than extrapolation from a generic hydrogel class.

## 7. Future Perspectives

A useful next step is to couple temperature-triggered gelation with a second response that addresses a defined limitation of local treatment. Temperature can govern depot formation, while pH, enzymes, ROS, or light regulate drug availability or antibacterial action [3,19,21]. The added response is most valuable when it improves a measurable outcome, such as maintaining activity during changing exposure conditions or reducing the dose needed for a given antibacterial effect. Feedback-responsive systems should therefore be evaluated as dynamic treatment strategies: infection-related signals change with bacterial burden and host repair, and the material response must remain effective as those signals evolve [19]. Comparing the responsive formulation with an otherwise matched nonresponsive formulation would help establish whether the extra function contributes enough benefit to justify its manufacturing and safety demands [3].

Future integrated functions include hemostasis, adhesion, immune modulation, and physical barriers, but each requires its own endpoint and controls. The postoperative-adhesion study of Zhan et al. illustrates a thermoresponsive physical barrier as a related research direction, without providing direct evidence of antibacterial efficacy [92]. Central-nervous-system delivery similarly raises tissue-mechanics and injection-injury questions before any antibacterial indication can be inferred [44]. AMPs, bacteriophages, and metal-containing systems may support antibiotic-sparing strategies, but the relevant dose reduction or treatment advantage must be demonstrated directly [53,62,73].

Three-dimensional bioprinting offers imaging-informed control of material placement and tissue geometry [93], while machine learning can assist materials screening and property prediction [94]. For antibacterial thermogels, these tools should address a defined formulation task, such as fitting an irregular defect or selecting compositions within a practical gelation window. Their application requires comparable training or design data and independent confirmation that processing preserves gelation, drug activity, release, and batch reproducibility. Translation should then connect material safety and representative animal studies with scalable manufacture and early clinical feasibility, including the intended use alongside debridement, revision surgery, or systemic therapy [45,85,91].

## 8. Conclusions

Injectable thermoresponsive hydrogels provide a route from a flowable precursor to a localized antibacterial depot. Across the studies considered here, their design is governed by the interaction of three factors: thermal behavior determines when and where the gel forms, carrier architecture influences drug availability, and anatomical conditions determine whether the formulation remains in contact with the target tissue [14,15,16,38]. Direct loading and particle-in-gel systems offer different ways to organize antibacterial and regenerative payloads, while contact-active and light-mediated components introduce additional exposure and safety requirements. The appropriate design therefore differs among a debrided bone cavity, an exuding wound, a periodontal pocket, and the ocular surface. In each setting, bacterial outcomes and tissue repair are complementary measures of treatment performance.

The immediate translational priority is to connect local residence and active-agent exposure with microbiological benefit, tissue compatibility, and eventual material clearance. This connection requires formulation-specific measurements under the relevant fluid, mechanical, and treatment conditions. Greater compositional complexity is justified when its benefit can be demonstrated against an appropriate simpler formulation. Reproducible manufacture and clinically meaningful outcomes will ultimately determine whether thermogel delivery provides a useful adjunct to infection management [45,56,85,91].

## Figures and Tables

**Figure 1:**
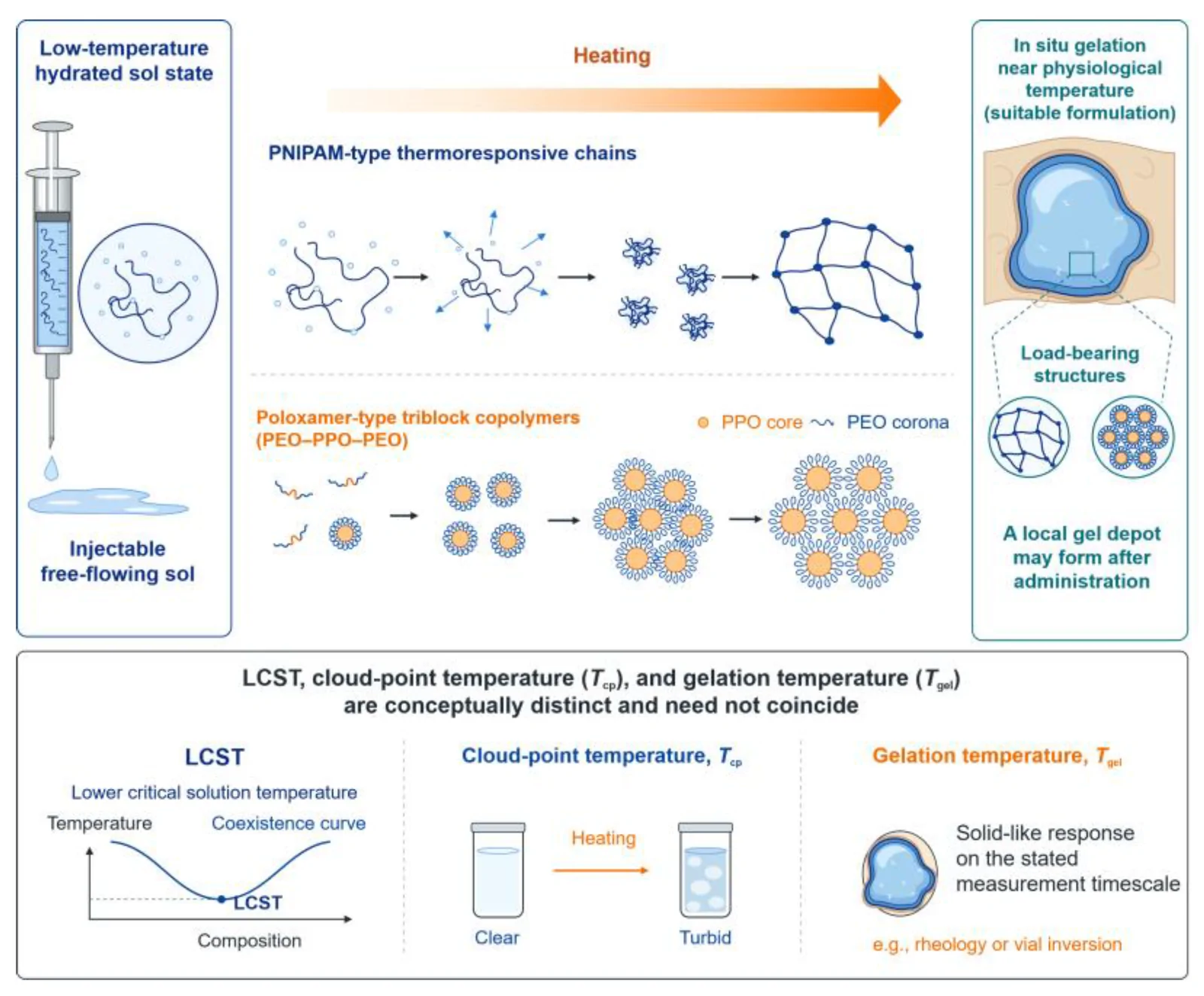
Distinct thermal pathways and the evidence required to identify a gel. PNIPAM-type dehydration or collapse and poloxamer micellization are separated. Neither turbidity nor the presence of a thermoresponsive polymer alone proves macroscopic gelation. Original conceptual schematic; no experimental data are plotted. PNIPAM, poly(N-isopropylacrylamide); CGT, critical gelation temperature. Blue polymer chains depict the PNIPAM-type pathway; orange cores and blue coronas depict the PPO and PEO regions of poloxamer micelles, respectively. Coils, micelles, and network symbols are schematic and are not drawn to scale. PPO, poly(propylene oxide); PEO, poly(ethylene oxide). This schematic was designed by the authors and graphically rendered with the assistance of ChatGPT (OpenAI). The scientific content, labels, and final composition were reviewed and verified by the authors.

**Figure 2:**
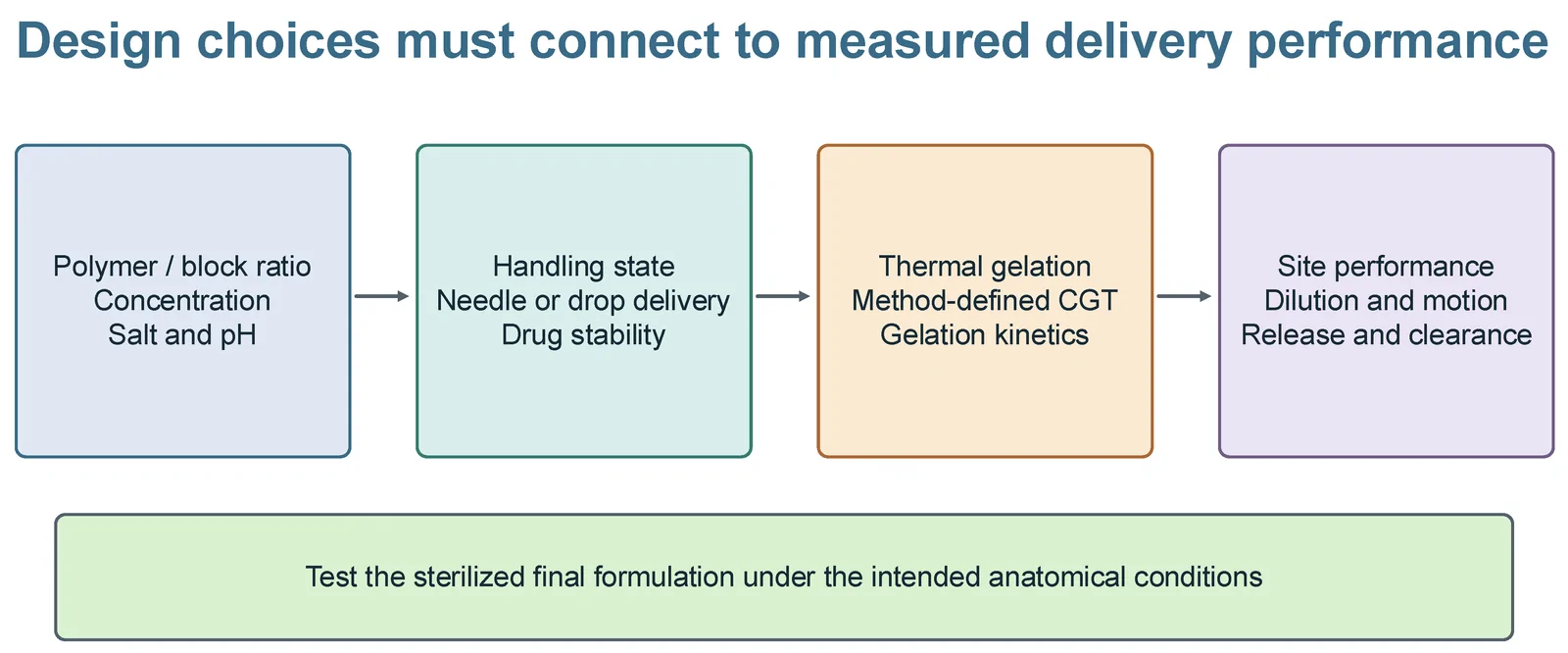
Formulation decisions connect administration, thermal gelation, and residence to measurable attributes. Injection force, gelation measurements, release, and clearance must be assessed under the intended route and tissue conditions. Original conceptual schematic. CGT, critical gelation temperature.

**Figure 3:**
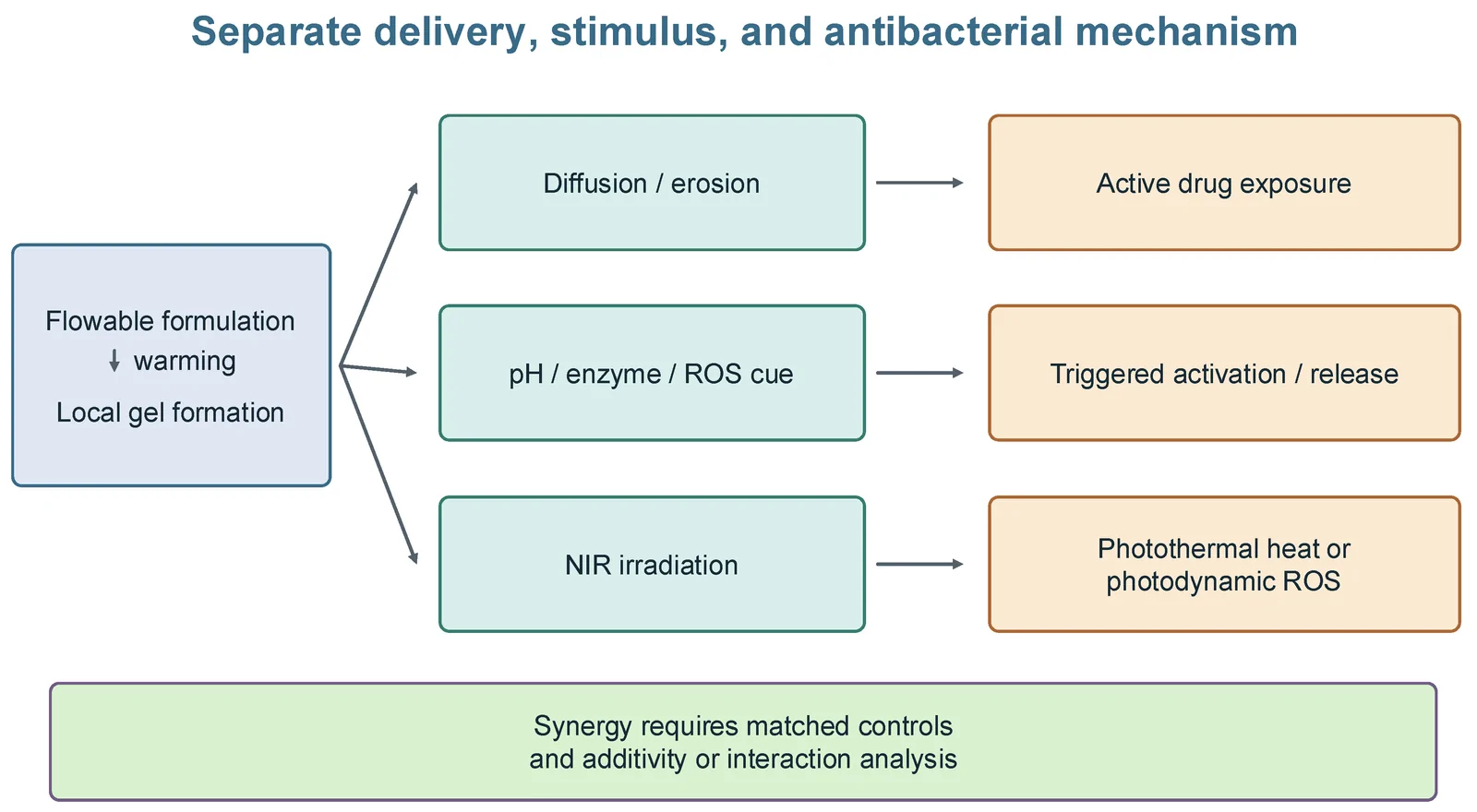
Design relationships in combined treatment. Thermal localization precedes release or secondary activation. NIR is an external stimulus, whereas photothermal conversion and photodynamic ROS generation are mechanisms. Synergy requires matched component and stimulus controls together with a prespecified additivity model or interaction analysis. Original conceptual schematic. NIR, near-infrared; ROS, reactive oxygen species. The downward arrow in the first box denotes warming-induced conversion of a flowable formulation into a local gel.

**Figure 4:**
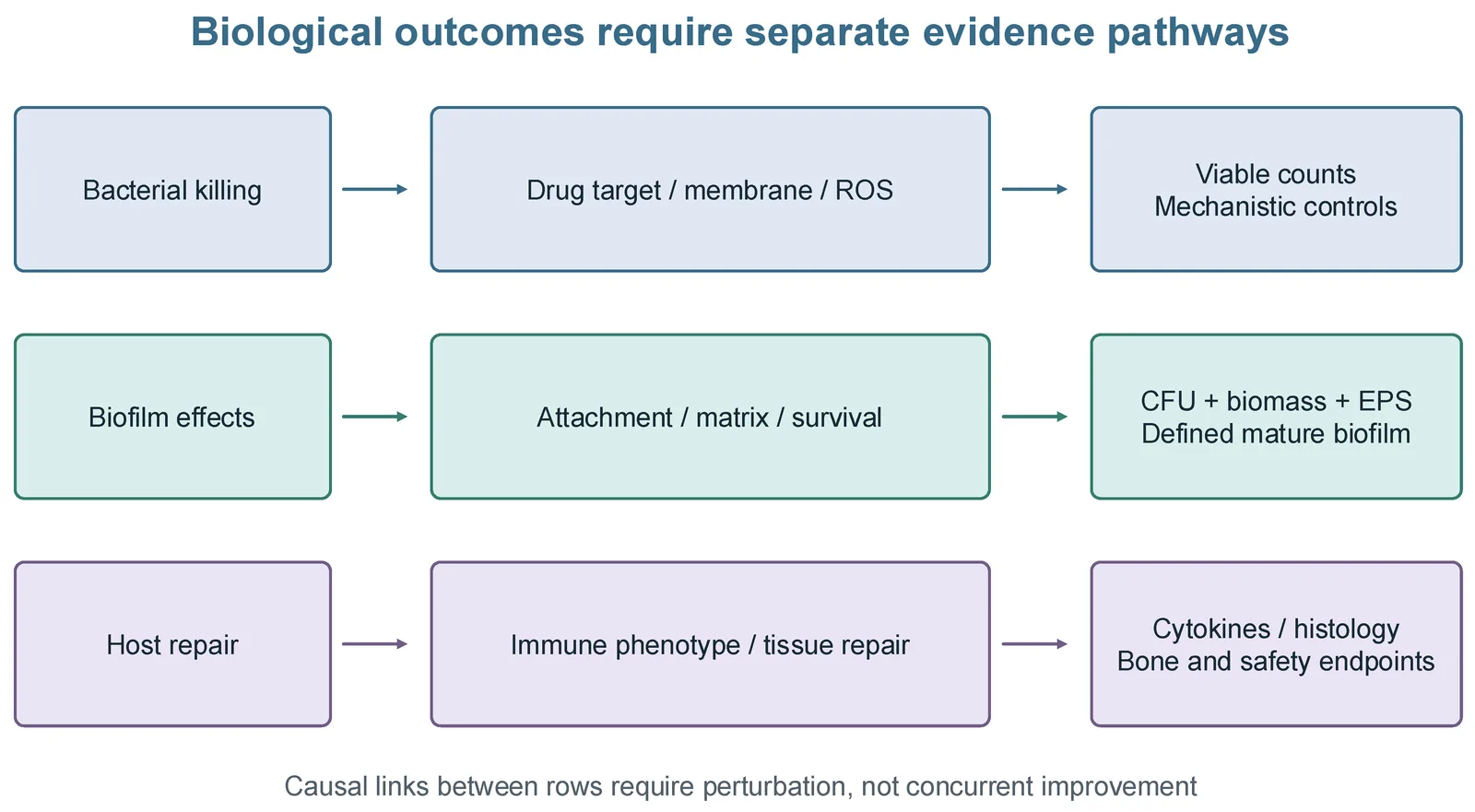
Separate evidence pathways for bacterial killing, biofilm effects, and host repair. Parallel outcomes do not prove a causal connection. Mechanistic arrows require perturbation and matched controls. Original conceptual schematic; assay examples are evaluation options, not results attributed to every cited study. CFU, colony-forming units; EPS, extracellular polymeric substances; ROS, reactive oxygen species.

**Figure 5:**
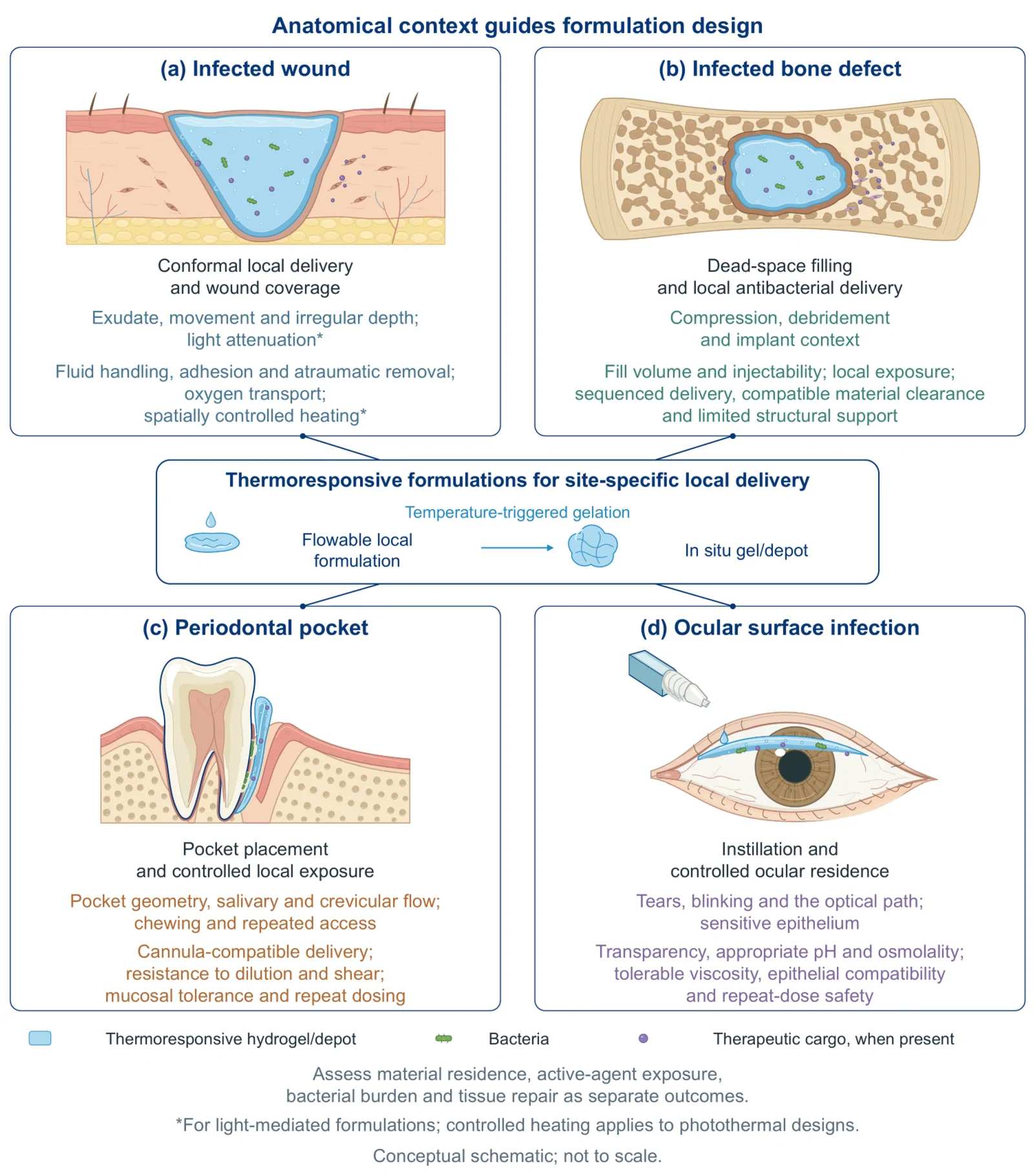
Anatomical constraints for the four principal application areas: infected wounds, infected bone defects, periodontal pockets, and ocular surfaces. Retention is tested against the relevant fluid flow and movement. Postoperative anti-adhesion barriers without antibacterial validation are excluded from the main antibacterial indication map. Original conceptual schematic. * Light attenuation applies to light-mediated formulations; spatially controlled heating applies to photothermal designs. Text colors distinguish the four application areas and do not encode quantitative values or evidence levels. This schematic was designed by the authors and graphically rendered with the assistance of ChatGPT (OpenAI). The scientific content, labels, and final composition were reviewed and verified by the authors.

**Table 6:** Controls and readouts needed to support antibacterial and host-response mechanisms.

Claim	Essential Controls	Minimum Complementary Readouts
Contact killing	Drug-free gel; free cationic agent; leachate; immobilized versus soluble agent.	CFU and membrane permeability; chemical analysis of leachables.
Metal-mediated killing	Blank gel; ion-only and particle-only controls; chelator-only plus chelator-treated formulation.	Total metal by ICP with separation/speciation for ion release; particle leakage; CFU; tissue/cell toxicity.
Photothermal killing	Light-only; material without light; external heat matched to the temperature–time exposure.	Irradiance and wavelength; temperature–time curve; viable counts; tissue injury.
ROS-mediated killing	Probe-interference controls; scavenger-only; non-ROS material; light and heat controls.	ROS assays with orthogonal confirmation; bacterial viability; host-cell toxicity.
Biofilm disruption	Prevention versus mature-biofilm treatment; untreated and free-drug controls; recovery controls.	Biofilm age and medium; recovered CFU; biomass; EPS and spatial viability imaging.
Immune-mediated repair	Blank gel; free drug; pathway inhibitor or depletion with its own control.	Macrophage markers and cytokines; histology; bacterial burden and bone/repair endpoints.
Combined-treatment synergy	All single components and stimulus combinations at matched dose.	Prespecified additivity model or interaction analysis; efficacy and toxicity together.

Note: CFU, colony-forming units; ICP, inductively coupled plasma; ROS, reactive oxygen species; EPS, extracellular polymeric substances.

**Table 7:** Clinical indication, anatomical constraints, required formulation attributes, and meaningful preclinical endpoints.

Indication	Anatomical Constraints	Required Formulation Attributes	Meaningful Endpoints
Infected wounds	Exudate, movement, irregular depth; light attenuation.	Fluid handling; adhesion and removal; oxygen transport; spatially controlled heating.	Wound CFU and recurrence; closure plus histology; tissue injury; residence during fluid exposure.
Infected bone defects	Dead space and compression; implants; debridement and structural support.	Fill volume and injectability; local exposure; release/repair sequence; compatible clearance.	Bone/implant cultures; recurrence; micro-CT and histology; material fate and mechanical support.
Periodontal pockets	Pocket geometry; saliva/crevicular flow; chewing; repeated access.	Cannula-compatible delivery; resistance to dilution/shear; mucosal tolerance; repeat dosing.	Pocket retention; biofilm viability; local exposure; alveolar bone and inflammatory endpoints.
Ocular surface infection	Tears, blinking, optical path; sensitive epithelium.	Transparent formulation; appropriate pH/osmolality; tolerable viscosity; controlled residence.	Corneal bacterial burden; opacity and epithelial integrity; tear clearance; repeat-dose safety.

## Data Availability

No new data were created or analyzed in this study. Data sharing is not applicable to this article.

## Abbreviations

AgNP, silver nanoparticle; ALP, alkaline phosphatase; AMP, antimicrobial peptide; BHI, brain heart infusion; β-GP, β-glycerophosphate; CFU, colony-forming units; CGT, critical gelation temperature; DGEA, Asp–Gly–Glu–Ala; EPS, extracellular polymeric substances; G′/G″, storage/loss modulus; ICP, inductively coupled plasma; LB, lysogeny broth; LCST, lower critical solution temperature; LDH, layered double hydroxide; LPS, lipopolysaccharide; MBC, minimum bactericidal concentration; MBEC, minimum biofilm eradication concentration; MIC, minimum inhibitory concentration; MOF, metal–organic framework; MRSA, methicillin-resistant *Staphylococcus aureus*; NIR, near-infrared; OCN, osteocalcin; OD, optical density; OPN, osteopontin; PCLGA, poly(ε-caprolactone-co-glycolide); PBS, phosphate-buffered saline; PDA, polydopamine; PEG, poly(ethylene glycol); PLGA, poly(lactic-co-glycolic acid); PMMA, poly(methyl methacrylate); PNIPAM, poly(N-isopropylacrylamide); POSS, polyhedral oligomeric silsesquioxane; ROS, reactive oxygen species; RUNX2, runt-related transcription factor 2; UI, uncertainty interval.

## References

[1] Murray C.J.L., Ikuta K.S., Sharara F., Swetschinski L., Robles Aguilar G., Gray A., Han C., Bisignano C., Rao P., Wool E. (2022). Global burden of bacterial antimicrobial resistance in 2019: A systematic analysis. Lancet.

[2] Blair J.M.A., Webber M.A., Baylay A.J., Ogbolu D.O., Piddock L.J.V. (2015). Molecular mechanisms of antibiotic resistance. Nat. Rev. Microbiol..

[3] Andresen A.K., Felding M.S., Sacramento M.M.A., Nielsen L.H. (2026). Hydrogel-based drug delivery systems for localized eradication of bacterial biofilms: Advances, challenges and future directions. J. Control. Release.

[4] Flemming H.C., Wingender J., Szewzyk U., Steinberg P., Rice S.A., Kjelleberg S. (2016). Biofilms: An emergent form of bacterial life. Nat. Rev. Microbiol..

[5] Holmes A.H., Moore L.S.P., Sundsfjord A., Steinbakk M., Regmi S., Karkey A., Guerin P.J., Piddock L.J.V. (2016). Understanding the mechanisms and drivers of antimicrobial resistance. Lancet.

[6] Wassif R.K., Elkayal M., Shamma R.N., Elkheshen S.A. (2021). Recent advances in the local antibiotics delivery systems for management of osteomyelitis. Drug Deliv..

[7] Hoare T.R., Kohane D.S. (2008). Hydrogels in drug delivery: Progress and challenges. Polymer.

[8] Lee K.Y., Mooney D.J. (2001). Hydrogels for tissue engineering. Chem. Rev..

[9] Buwalda S.J., Boere K.W.M., Dijkstra P.J., Feijen J., Vermonden T., Hennink W.E. (2014). Hydrogels in a historical perspective: From simple networks to smart materials. J. Control. Release.

[10] Wang Y., Wang Z., Lu W., Hu Y. (2024). Review on chitosan-based antibacterial hydrogels: Preparation, mechanisms, and applications. Int. J. Biol. Macromol..

[11] Lan X., Du T., Zhuo J., Wang T., Shu R., Li Y., Zhang W., Ji Y., Wang Y., Yue X. (2024). Advances of biomacromolecule-based antibacterial hydrogels and their performance evaluation for wound healing: A review. Int. J. Biol. Macromol..

[12] Li S., Dong S., Xu W., Tu S., Yan L., Zhao C., Ding J., Chen X. (2018). Antibacterial hydrogels. Adv. Sci..

[13] Li Y., Han Y., Li H., Niu X., Zhang D., Wang K. (2024). Antimicrobial hydrogels: Potential materials for medical application. Small.

[14] Zhang K., Xue K., Loh X.J. (2021). Thermo-responsive hydrogels: From recent progress to biomedical applications. Gels.

[15] Xiao Y., Gu Y., Qin L., Chen L., Chen X., Cui W., Li F., Xiang N., He X. (2021). Injectable thermosensitive hydrogel-based drug delivery system for local cancer therapy. Colloids Surf. B Biointerfaces.

[16] Tao J., Zhang Y., Shen A., Yang Y., Diao L., Wang L., Cai D., Hu Y. (2020). Injectable chitosan-based thermosensitive hydrogel/nanoparticle-loaded system for local delivery of vancomycin in the treatment of osteomyelitis. Int. J. Nanomed..

[17] Zheng Y., Wang Y., Sheng F., Wang S., Zhou Y., Li X., Li N., Song W., Song Z. (2024). Treatment of chronic osteomyelitis with gradient release of DGEA and vancomycin hydrogel-microsphere system and its mechanism. Front. Pharmacol..

[18] Yu X., Liu H., Chen L., Cheng X., Wu G., Forouzanfar T., Feng L., Guo R., Zhou M., Sun T. (2026). Thermosensitive antibacterial nanocomposite hydrogel guiding macrophage polarization and bone regeneration for periodontitis treatment. Bioact. Mater..

[19] Yang Y., Cui Y., Wu Q., Tai Z., Zhu Q., Chen Z., Wu W., Lu Y. (2025). Cutting-edge hydrogel systems with intrinsic functions: Redefining chronic infection treatment. Chem. Eng. J..

[20] Salehi S., Naghib S.M., Garshasbi H.R., Ghorbanzadeh S., Zhang W. (2023). Smart stimuli-responsive injectable gels and hydrogels for drug delivery and tissue engineering applications: A review. Front. Bioeng. Biotechnol..

[21] Sun S., Jiang G., Dong J., Xie X., Liao J., Tian Y. (2024). Photothermal hydrogels for infection control and tissue regeneration. Front. Bioeng. Biotechnol..

[22] Chen H., Xu J., Sun J., Jiang Y., Zheng W., Hu W., Qian H. (2024). Recent advances on thermosensitive hydrogels-mediated precision therapy. Asian J. Pharm. Sci..

[23] Hao Z., Li X., Zhang R., Zhang L. (2024). Stimuli-responsive hydrogels for antibacterial applications. Adv. Healthc. Mater..

[24] Di Martino M., Sessa L., Pagliari G., Silvestrino D., Concilio S. (2026). Injectable Thermoresponsive Hydrogels for Localized Drug Delivery: Mechanisms, In Vivo Evidence and Translational Challenges. J. Funct. Biomater..

[25] Gou X., Cheng Y., Zhang C., Xie J., Chen B., Chen Y., Zhang S., Ma G. (2026). Phase transition mechanism and research progress of LCST-type thermosensitive hydrogels. Macromol. Rapid Commun..

[26] Schild H.G. (1992). Poly(N-isopropylacrylamide): Experiment, theory and application. Prog. Polym. Sci..

[27] Pelton R. (2000). Temperature-sensitive aqueous microgels. Adv. Colloid Interface Sci..

[28] Tang L., Wang L., Yang X., Feng Y., Li Y., Feng W. (2021). Poly(N-isopropylacrylamide)-based smart hydrogels: Design, properties and applications. Prog. Mater. Sci..

[29] Overstreet D.J., Dutta D., Stabenfeldt S.E., Vernon B.L. (2012). Injectable hydrogels. J. Polym. Sci. Part B Polym. Phys..

[30] Narayana S., Gowda B.H.J., Hani U., Ahmed M.G., Asiri Z.A., Paul K. (2025). Smart poly(N-isopropylacrylamide)-based hydrogels: A tour d’horizon of biomedical applications. Gels.

[31] Marques A.C., Costa P.C., Velho S., Amaral M.H. (2023). Injectable poloxamer hydrogels for local cancer therapy. Gels.

[32] Liow S.S., Dou Q., Kai D., Karim A.A., Zhang K., Xu F., Loh X.J. (2016). Thermogels: In situ gelling biomaterial. ACS Biomater. Sci. Eng..

[33] Moon S.H., Park S.J., Lee Y.W., Yang Y.J. (2024). LCST/UCST behavior of polysaccharides for hydrogel fabrication. RSC Adv..

[34] Huang Y., Guo W., Zhang J., Peng X., Li G., Zhang L.M., Yang L. (2020). Thermosensitive hydrogels based on methylcellulose derivatives for prevention of postoperative adhesion. Cellulose.

[35] Hiruta Y. (2022). Poly(*N*-isopropylacrylamide)-based temperature- and pH-responsive polymer materials for application in biomedical fields. Polym. J..

[36] Ansari M.J., Rajendran R.R., Mohanto S., Agarwal U., Panda K., Dhotre K., Manne R., Deepak A., Zafar A., Yasir M. (2022). Poly(N-isopropylacrylamide)-based hydrogels for biomedical applications: A review of the state-of-the-art. Gels.

[37] Chen Z., Wang A., Qin Y., Chen X., Feng X., He G., Zhu X., Xiao Y., Yu X., Zhong T. (2024). Preparation of a thermosensitive and antibacterial in situ gel using poloxamer-quaternized chitosan for sustained ocular delivery of levofloxacin hydrochloride. Int. J. Biol. Macromol..

[38] Yuan B., Zhang Y., Wang Q., Ren G., Wang Y., Zhou S., Wang Q., Peng C., Cheng X. (2022). Thermosensitive vancomycin@PLGA-PEG-PLGA/HA hydrogel as an all-in-one treatment for osteomyelitis. Int. J. Pharm..

[39] Zhi C., Wang G., Zhou B., Liu P., Kang Y., Chen W. (2026). Injectable butyl glycidyl ether-modified methylcellulose thermosensitive hydrogel for topical delivery of vancomycin hydrochloride in the treatment of osteomyelitis. J. Biomed. Mater. Res. Part A.

[40] Hennink W.E., van Nostrum C.F. (2012). Novel crosslinking methods to design hydrogels. Adv. Drug Deliv. Rev..

[41] Gong J.P. (2010). Why are double network hydrogels so tough?. Soft Matter.

[42] Wyszogrodzka G., Marszałek B., Gil B., Dorożyński P. (2016). Metal-organic frameworks: Mechanisms of antibacterial action and potential applications. Drug Discov. Today.

[43] Ahmed E.M. (2015). Hydrogel: Preparation, characterization, and applications: A review. J. Adv. Res..

[44] Bellotti E., Schilling A.L., Little S.R., Decuzzi P. (2021). Injectable thermoresponsive hydrogels as drug delivery system for the treatment of central nervous system disorders: A review. J. Control. Release.

[45] Tanga S., Aucamp M., Ramburrun P. (2023). Injectable thermoresponsive hydrogels for cancer therapy: Challenges and prospects. Gels.

[46] Kai K.C., Borges R., Pedroni A.C.F., Pelosine A.M., da Cunha M.R., Marques M.M., de Araújo D.R., Marchi J. (2024). Tricalcium phosphate-loaded injectable hydrogel as a promising osteogenic and bactericidal teicoplanin-delivery system for osteomyelitis treatment: An in vitro and in vivo investigation. Biomater. Adv..

[47] Shim G.Y., Moon S.H., Shin S.J., Kim H.J., Oh S., Bae J.M. (2025). Emodin-loaded thermoresponsive hydrogel as a potential drug delivery system for periodontal disease in a rat model of ligature-induced periodontitis. Polymers.

[48] Aghamirsalim M., Mobaraki M., Jabbarvand M., Sahraian A. (2026). Thermosensitive chitosan–hyaluronic acid hydrogel for sustained betamethasone and levofloxacin delivery in corneal wound healing. Transl. Vis. Sci. Technol..

[49] Zheng L., Chen Y., Han Y., Lin J., Fan K., Wang M., Teng T., Yang X., Ke L., Li M. (2024). Thermosensitive polyhedral oligomeric silsesquioxane hybrid hydrogel enhances the antibacterial efficiency of erythromycin in bacterial keratitis. Biomater. Res..

[50] Zheng W., Lin M., Cheng H., Gao X., Xiang Y., Wan W., Zheng S., Huang X., Hu K. (2025). A multifunctional hydrogel system with synergistic effects against *Staphylococcus aureus* infections and inflammation in perioperative cataract surgery. Mater. Today Bio.

[51] Ge P., Chang S., Wang T., Zhao Q., Wang G., He B. (2023). An antioxidant and antibacterial polydopamine-modified thermo-sensitive hydrogel dressing for *Staphylococcus aureus*-infected wound healing. Nanoscale.

[52] Lew D.P., Waldvogel F.A. (2004). Osteomyelitis. Lancet.

[53] Lemire J.A., Harrison J.J., Turner R.J. (2013). Antimicrobial activity of metals: Mechanisms, molecular targets and applications. Nat. Rev. Microbiol..

[54] Albao M.J.F., Calsis J.R.F., Dancel J.O., De Juan-Corpuz L.M., Corpuz R.D. (2025). Silver nanoparticle-infused hydrogels for biomedical applications: A comprehensive review. J. Chin. Chem. Soc..

[55] Huh A.J., Kwon Y.J. (2011). “Nanoantibiotics”: A new paradigm for treating infectious diseases using nanomaterials in the antibiotics resistant era. J. Control. Release.

[56] Chernousova S., Epple M. (2013). Silver as antibacterial agent: Ion, nanoparticle, and metal. Angew. Chem. Int. Ed..

[57] Rai M., Yadav A., Gade A. (2009). Silver nanoparticles as a new generation of antimicrobials. Biotechnol. Adv..

[58] Ikwuagwu P.A., Chen A., Pang W., He X., Gao C., Wu A., Xu C. (2026). Thermosensitive eye drop loaded with copper-based layered double hydroxide nanosheets for treatment of bacterial keratitis. Colloids Surf. A Physicochem. Eng. Asp..

[59] Zasloff M. (2002). Antimicrobial peptides of multicellular organisms. Nature.

[60] Hancock R.E.W., Sahl H.G. (2006). Antimicrobial and host-defense peptides as new anti-infective therapeutic strategies. Nat. Biotechnol..

[61] Brogden K.A. (2005). Antimicrobial peptides: Pore formers or metabolic inhibitors in bacteria?. Nat. Rev. Microbiol..

[62] Mookherjee N., Anderson M.A., Haagsman H.P., Davidson D.J. (2020). Antimicrobial host defence peptides: Functions and clinical potential. Nat. Rev. Drug Discov..

[63] Cai D., Li C., Zhu T., Li R., Zhang M., Li X., Liu Y., Dai Z., Wan L., Lu H. (2025). Antimicrobial peptide hydrogels: Synthesis, ROS regulation mechanism, and multimodal therapeutic applications in drug delivery systems. J. Mater. Chem. B.

[64] Feng T., Wu H., Ma W., Wang Z., Wang C., Wang Y., Wang S., Zhang M., Hao L. (2022). An injectable thermosensitive hydrogel with a self-assembled peptide coupled with an antimicrobial peptide for enhanced wound healing. J. Mater. Chem. B.

[65] Ma S., Lu X., Yu X., Du Y., Xu S., Li M., Peng C., Liu Z., Deng J. (2022). An injectable multifunctional thermo-sensitive chitosan-based hydrogel for periodontitis therapy. Biomater. Adv..

[66] Li T., Wang P., Guo W., Huang X., Tian X., Wu G., Xu B., Li F., Yan C., Liang X.J. (2019). Natural berberine-based Chinese herb medicine assembled nanostructures with modified antibacterial application. ACS Nano.

[67] Liu D., Chen J., Gao L., Chen X., Lin L., Wei X., Liu Y., Cheng H. (2024). Injectable photothermal PDA/chitosan/β-glycerophosphate thermosensitive hydrogels for antibacterial and wound healing promotion. Macromol. Biosci..

[68] Huang Z., Ji C., Zang X. (2026). Temperature- and pH-responsive near-infrared-activated hydrogel with synergistic antibacterial activity. J. Appl. Polym. Sci..

[69] Nathan C., Cunningham-Bussel A. (2013). Beyond oxidative stress: An immunologist’s guide to reactive oxygen species. Nat. Rev. Immunol..

[70] Davies D. (2003). Understanding biofilm resistance to antibacterial agents. Nat. Rev. Drug Discov..

[71] Hall-Stoodley L., Costerton J.W., Stoodley P. (2004). Bacterial biofilms: From the natural environment to infectious diseases. Nat. Rev. Microbiol..

[72] Bjarnsholt T., Alhede M., Alhede M., Eickhardt-Sørensen S.R., Moser C., Kühl M., Jensen P.Ø., Høiby N. (2013). The in vivo biofilm. Trends Microbiol..

[73] Koo H., Allan R.N., Howlin R.P., Stoodley P., Hall-Stoodley L. (2017). Targeting microbial biofilms: Current and prospective therapeutic strategies. Nat. Rev. Microbiol..

[74] Murray P.J., Wynn T.A. (2011). Protective and pathogenic functions of macrophage subsets. Nat. Rev. Immunol..

[75] Eming S.A., Martin P., Tomic-Canic M. (2014). Wound repair and regeneration: Mechanisms, signaling, and translation. Sci. Transl. Med..

[76] Gurtner G.C., Werner S., Barrandon Y., Longaker M.T. (2008). Wound repair and regeneration. Nature.

[77] Martin P. (1997). Wound healing: Aiming for perfect skin regeneration. Science.

[78] Sen C.K., Gordillo G.M., Roy S., Kirsner R., Lambert L., Hunt T.K., Gottrup F., Gurtner G.C., Longaker M.T. (2009). Human skin wounds: A major and snowballing threat to public health and the economy. Wound Repair Regen..

[79] Armstrong D.G., Boulton A.J.M., Bus S.A. (2017). Diabetic foot ulcers and their recurrence. N. Engl. J. Med..

[80] Boulton A.J.M., Vileikyte L., Ragnarson-Tennvall G., Apelqvist J. (2005). The global burden of diabetic foot disease. Lancet.

[81] Gou Y., Wang A., Liu J., Ding B., Song W., Sun Q., Qu W., Zhou X., Jiang L., Guo H. (2026). Thermo-responsive mechanically active hydrogel based on dual dynamic covalent cross-linked with antibacterial and antioxidant activity for wound healing. Chem. Eng. Sci..

[82] Gong T., Feng R., Zhu H., Zhao M., Yu B., Wang N., Wang H., Guo R. (2026). Multi-functional poly(N-isopropylacrylamide)-based hydrogel with hemostasis, antibacterial, and sustained drug release properties for infected wound treatment. Colloids Surf. B Biointerfaces.

[83] Xu P., Xiong J., Cheng B., Wang R., Ma X., Wu X., Zhang B., Chen Z., Chen Q., Zhao G. (2026). Dual crosslinked thermosensitive hydrogel engineered with multiple dynamic hydrogen bonds for enhanced photodynamic antibacterial activity and wound healing. Acta Biomater..

[84] Irani A.E., Farahpour M.R., Valilou M.R., Tabatabaei Z.G. (2026). Accelerating the healing of infected full thickness excision wounds through the topical use of Pluronic F127 copolymer and Polyglutamic acid. Injury.

[85] Tande A.J., Patel R. (2014). Prosthetic joint infection. Clin. Microbiol. Rev..

[86] Cao Z., Qin Z., Duns G.J., Huang Z., Chen Y., Wang S., Deng R., Nie L., Luo X. (2024). Repair of infected bone defects with hydrogel materials. Polymers.

[87] Wang M., Zheng Y., Yin C., Dai S., Fan X., Jiang Y., Liu X., Fang J., Yi B., Zhou Q. (2023). Recent progress in antibacterial hydrogel coatings for targeting biofilm to prevent orthopedic implant-associated infections. Front. Microbiol..

[88] Pihlstrom B.L., Michalowicz B.S., Johnson N.W. (2005). Periodontal diseases. Lancet.

[89] Hajishengallis G. (2015). Periodontitis: From microbial immune subversion to systemic inflammation. Nat. Rev. Immunol..

[90] Urtti A. (2006). Challenges and obstacles of ocular pharmacokinetics and drug delivery. Adv. Drug Deliv. Rev..

[91] Hoffman A.S. (2013). Stimuli-responsive polymers: Biomedical applications and challenges for clinical translation. Adv. Drug Deliv. Rev..

[92] Zhan Y., Zhao X., He C., Bi S., Liu R., Gu J., Yan B. (2025). Temperature-responsive and self-healing hydrogel: A novel approach to combat postoperative adhesions. Polymers.

[93] Murphy S.V., Atala A. (2014). 3D bioprinting of tissues and organs. Nat. Biotechnol..

[94] Butler K.T., Davies D.W., Cartwright H., Isayev O., Walsh A. (2018). Machine learning for molecular and materials science. Nature.

